# Assessing the adverse effects of COVID-19 vaccine in different scenarios in Saudi Arabia: A cross-sectional study

**DOI:** 10.15537/smj.2023.44.2.20220680

**Published:** 2023-02

**Authors:** Ahad Amer Alsaiari, Mamdouh Allahyani, Abdulelah Aljuaid, Alaa Shafie, Ayman Al-hazmi, Haytham A. Dahlawi, Osama Abdulaziz, Ebtisam Alosimi, Albatool Alzaidi, Mazen Almehmadi

**Affiliations:** *From the Department of Clinical Laboratory Sciences, College of Applied Medical Sciences, Taif University, Taif, Kingdom of Saudi Arabia*

**Keywords:** COVID-19, vaccines, side effect, symptoms, Saudi Arabia

## Abstract

**Objectives:**

To assess the different side effects of COVID-19 vaccines at different scenarios in Saudi Arabia.

**Methods:**

This cross-sectional study sought to investigate the side effects of COVID-19 vaccines through an online survey of 2,718 participants in Saudi Arabia.

**Results:**

People can manage their expectations about vaccine side effects and deal with symptoms better by knowing beforehand that they are likely to experience mild side effects for a short period, symptoms that are manifested regardless of age, and infection before or after vaccination. There are certain uncommon side effects that affect more people who got infected, and not before vaccination; there are side effects that disproportionately impact women, and also the side effects that wane after the second dose.

**Conclusion:**

These findings can assist in evaluating the concerns regarding vaccine acceptance. The public should be made aware that they are likely to experience at least one side effect, with temporary post-injection inflammation, musculoskeletal pain, fever, and headache as the most commonly reported side effects across the board. However, the common symptoms are mild to moderate, and the side effects last for a short period for most people.

**H**ealthcare services around the world have been significantly impacted by the COVID-19 pandemic and its rapid spread around the world.^1-3^ Nevertheless, many individuals who are infected with the virus are asymptomatic, which means they can still transmit the infection. Thus, preventing the spread of the virus throughout communities is incredibly challenging. People around the world have been striving to find solutions to the pandemic by vaccinating their populations in order to eliminate the disease.^4-6^ Coronavirus vaccines encourage the immune system to produce antibodies against the virus, which ultimately protects individuals from becoming infected or developing severe symptoms.^3,7-9^ Following the vaccination, the produced antibodies stick to the invading spikes as proteins and stop the virus from entering the cells.^10^

At present, there are 4 coronavirus vaccines that have been approved for administration around the world, namely: BNT162 (Pfizer BioNTech, New York, NY, USA), ChAdOx1 (AstraZeneca, Oxford, UK), mRNA1273 (Moderna, Cambridge, MA, USA), and Ad26.COV2-S (Johnson & Johnson, New Brunswick, NJ, USA). Moreover, several other vaccines have been developed for use in specific countries, such as BBIBP-CorV (Sinopharm, Beijing, China), CoronaVac (Sinovac, Beijing, China), Sputnik V (Gamaleya, Moscow, Russian), and COVAXIN (Bharat Biotech, Hyderabad, India).^9^

The development of immunity after vaccination can occasionally cause side effects. These side effects are most likely the main cause of vaccine hesitancy among the population.^11^ Once vaccinated, a majority of individuals become immune to coronavirus, irrespective of any side effects. An investigation carried out by Elgendy et al^12^ revealed that just one in people suffer side effects from the coronavirus vaccination, and most such effects are both mild and short-lived.

The aim of this study is to assess the different side effects of COVID-19 vaccines at different scenarios in Saudi Arabia.

## Methods

This cross-sectional study with adequate sample size was performed through Google forms, a questionnaire through which different questions were sent to the participants via social media. The response was great as many people were satisfied to share their experience with the immunization. The link for the questionnaire was distributed from September 2021 to May 2022. The number of participants were 2,718 and introduced to the research, the researcher’s e-mail. Google scholar, PubMed, and web of sciences were used to find related literature to our work. Exclusion of any carried out.

This study has been approved by the Research Ethics Committee at Taif University, Taif, Saudi Arabia, and has received the approval number (HAO-02-T-105).

### Data analysis

After the number of participants reached our target sample size, the answers were exported into and excel file, which were then analyzed by using GNU PSPP 0.10.1-g1082b8 (PSPP Inc., Chicago, IL, USA). Answers were expressed as frequencies and percentages for every type of question, for example, about groups of age or gender. They were then compared by applying Pearson’s Chi-square test and a *p*-value of 0.05 was considered significant.

## Results

A total of 2,718 participants were engaged in this study from different regions in Saudi Arabia. Thirty-one (1%) had not received any vaccination dose, therefore they have been excluded from this study (Table 1). Of the 2,687 participants who received a vaccine, 1,583 (59%) were female and 1,104 (41%) were male (Table 2). The participants varied in terms of age range, 20% were ≤20 years old, 45% were between 21-35 years old, 25%, were between 36-50 years old, 8% were between 51-65 years old, and 1% were >65 years old (Table 3). Tables 1 to 6 show the most commonly reported side effects together with the side effects related to gender, age, infection before vaccination, infection after vaccination, and duration of the effects.

**Table 1:** - Common vaccine side effects related to participants’ infection status before vaccination.

Vaccine side effects	Yes	No	Total	*P*-value
Hospitalized due to severe symptoms	10	13	23	0.295
Severe symptoms but not hospitalized	79	160	239	0.510
Long-lasting post-injection inflammation	50	79	129	0.137
Temporary post-injection inflammation	126	368	494	0.019
Fever	133	275	408	0.735
Musculoskeletal pain	148	335	483	0.984
Sore throat	25	33	58	0.105
Congestion	13	27	40	0.916
Diarrhea	11	15	26	0.456
Conjunctivitis	6	8	14	0.235
Headache	93	207	300	0.197
Anosmia	15	7	22	0.001
Ageusia	14	7	21	0.002
Difficulty in communicating	1	9	10	0.344
Difficulty in movement	35	72	107	0.883
Allergy and rash	6	8	14	0.050
Dyspnea	10	26	36	0.262
Chest pain	18	25	43	0.170
No symptoms	41	184	225	0.001
Total	834	1858	2692	

*P*-value of 0.05 was considered significant.

**Table 2:** - Vaccine side effects related to participants’ gender.

Vaccine side effects	Male	Female	Total	*P*-value
Hospitalized due to severe symptoms	7 (30)	16 (70)	23	0.506
Severe symptoms but not hospitalized	100 (42)	139 (58)	239	0.879
Long-lasting post-injection inflammation	45 (35)	84 (65)	129	0.359
Temporary post-injection inflammation	194 (39)	300 (61)	494	0.599
Fever	182 (45)	226 (55)	408	0.354
Musculoskeletal pain	196 (41)	287 (59)	483	0.885
Sore throat	17 (29)	41 (71)	58	0.243
Congestion	15 (38)	25 (63)	40	0.768
Diarrhea	10 (38)	16 (62)	26	0.862
Conjunctivitis	4 (29)	10 (71)	14	0.542
Headache	109 (36)	191 (64)	300	0.283
Anosmia	7 (32)	15 (68)	22	0.571
Ageusia	8 (38)	13 (62)	21	0.858
Difficulty in communicating	0 (0)	5 (100)	5	0.231
Difficulty in movement	37 (35)	70 (65)	107	0.201
Allergy and rash	7 (50)	7 (50)	14	0.664
Dyspnea	6 (17)	30 (83)	36	0.056
Chest pain	13 (30)	30 (70)	43	0.354
No symptoms	147 (65)	78 (35)	225	0.001
Total	1104 (41)	1583 (59)	2687	

Values are presented as number and percentages (%). *P*-value of 0.05 was considered significant.

**Table 3:** - Vaccine side effects related to the participant’s age.

Vaccine side effects	≤20	21-35	36-50	51-65	>65	Total	*P*-value
Hospitalized due to severe symptoms	5 (22)	9 (39)	9 (39)	0 (0)	0 (0)	23	0.384
Severe symptoms but not hospitalized	43 (18)	119 (50)	59 (25)	17 (7)	1 (0)	239	0.586
Long-lasting post-injection inflammation	30 (23)	57 (44)	33 (26)	9 (7)	0 (0)	129	0.680
Temporary post-injection inflammation	86 (17)	208 (42)	132 (27)	62 (13)	6 (1)	494	0.008
Fever	75 (18)	185 (45)	108 (26)	33 (8)	7 (2)	408	0.568
Musculoskeletal pain	95 (20)	229 (47)	120 (25)	34 (7)	5 (1)	483	0.821
Sore throat	14 (24)	26 (45)	16 (28)	2 (3)	0 (0)	58	0.578
Congestion	4 (10)	22 (55)	13 (33)	1 (3)	0 (0)	40	0.219
Diarrhea	2 (8)	16 (62)	7 (27)	1 (4)	0 (0)	26	0.353
Conjunctivitis	4 (29)	4 (29)	6 (43)	0 (0)	0 (0)	14	0.351
Headache	70 (23)	155 (52)	59 (20)	13 (4)	3 (1)	300	0.008
Anosmia	5 (23)	10 (45)	6 (27)	1 (5)	0 (0)	22	0.948
Ageusia	4 (16)	11 (44)	5 (20)	5 (20)	0 (0)	25	0.312
Difficulty in communicating	1 (20)	1 (20)	2 (40)	0 (0)	1 (20)	5	0.001
Difficulty in movement	32 (30)	43 (40)	28 (26)	4 (4)	0 (0)	107	0.046
Allergy and rash	3 (21)	8 (57)	2 (14)	1 (7)	0 (0)	14	0.867
Dyspnea	13 (36)	11 (31)	11 (31)	1 (3)	0 (0)	36	0.075
Chest pain	9 (21)	15 (35)	16 (37)	3 (7)	0 (0)	43	0.397
No symptoms	44 (20)	90 (41)	44 (20)	38 (17)	4 (2)	220	0.001
Total	539 (20)	1219 (45)	676 (25)	225 (8)	27 (1)	2686	

Values are presented as number and percentages (%). *P*-value of 0.05 was considered significant.

Table 1 shows the vaccine side effects reported by participants who were infected or not infected before vaccination. More respondents were not infected (69%) before vaccination compared to those who were infected (31%) before vaccination. Most of the respondents (91.6%) who were infected or not infected before vaccination reported vaccine side effects. The most common vaccine side effects were the same for the participants who got infected and did not get infected before vaccination. Of the 834 participants who were infected before vaccination, the most commonly reported vaccine side effects were musculoskeletal pain (17.8%), fever (15.9%), temporary post-injection inflammation (15.1%), and headache (11.2%). Of the 1,858 participants who did not get infected before vaccination, the most common vaccine side effects were temporary post-injection inflammation (19.81%), musculoskeletal pain (18%), fever (14.80%), and headache (11.1%). The intensity of the most common vaccine side effects reported were mild to moderate. Between those who got infected and those who were not infected before vaccination, statistically significant differences in reported side effects emerged for temporary post-injection inflammation (*p*=0.019), anosmia (*p*=0.001), ageusia (*p*=0.002), and allergy and rash (*p*=0.050). Significantly more people who got infected before vaccination experienced anosmia and ageusia as side effects, while significantly more people who did not get infected before vaccination experienced temporary post-injection inflammation and allergy and rash. Between the 2 groups, many, who did not get infected before vaccination, reported severe symptoms. Although 1% of participants reported hospitalization due to severe symptoms as a vaccine side effect, slightly more participants who did not get infected before vaccination reported this side effect (56.5%) when compared to those who got infected (43.5%) before vaccination. Moreover, 8.9% who reported severe symptoms without hospitalization as a vaccine side effect, a greater number of participants (67%) who did not get infected before vaccination reported this side effect when compared to participants who got infected (33%) before vaccination. A significant number of participants (8.4%, *p*=0.000) reported absolutely no symptoms. However, participants (9.9%) who did not get infected before vaccination reported no vaccine symptoms when compared to participants (41, 4.9%) who got infected before vaccination. Although the participants who were infected or not infected before vaccination reported the same common side effects, there are certain side effects reported more by those infected before vaccination that significantly varied from those reported more by those not infected before vaccination. Since more respondents were not infected before vaccination, hospitalization due to severe symptoms and severe symptoms but not hospitalization were higher for this group. Significantly more participants who did not get infected before vaccination also reported no symptoms.

Table 2 shows vaccine side effects according to gender. The majority of male and female (91.6%) participants reported to have experienced some side effects. However, more females (95.1%) reported vaccine side effects when compared to males (86.7%). There are no other specific differences in the most common side effects reported by males and females. The most common side effects are musculoskeletal pain (41% males; 59% females), temporary post-injection inflammation (39% males; 61% females), fever (182, 45% males; 226, 55% females), headache (109, 36% males; 191, 64% females), and severe symptoms but not hospitalization (142% males; 58% females). Temporary post-injection inflammation is the leading side effect among females as opposed to musculoskeletal pain for males. The other 3 commonly reported side effects rank similarly for males and females. Regarding the severity of symptoms, more females (70%) than males (30%) reported hospitalization due to severe symptoms, and more females (58%) than males (42%) reported severe symptoms but not hospitalization. Concurrently, there is also a statistically significant variance between males and females in terms of those who reported no symptoms (65% males; 5% females; *p*=0.000). Gender is a determinant of experiencing and not experiencing any vaccine side effects with significantly more females experiencing symptoms and significantly more males not experiencing symptoms following vaccination.

Table 3 shows vaccine side effects related to the age of participants. Most of the participants across all age groups reported side effects (91.8%). The most common side effects across age groups are temporary post-injection inflammation, fever, musculoskeletal pain, and headache. Relative to specific side effects across age groups, statistical differences emerged in 4 side effects. A highly statistically significant (*p*=0.001) difference exists across age groups for the symptom of hard to talk, with 2 or 40% of the 36-50 age group reporting this side effect. Variance in reported temporary post-injection inflammation (*p*=0.008) and headache (*p*=0.008) also emerged across age groups. Nearly half (42%) of those who reported experiencing temporary post-injection inflammation belonged to the 21-35 age group. The majority (52%) of those who reported headache as a side effect belonged to the 21-35 age group. A statistically significant difference also emerged across age groups in reporting difficulty in movement as a side effect, with most participants reporting this symptom falling under the ≤20 age group (30%), 21-35 age group (40%), and 36-50 age group (26%). A highly statistically significant (*p*=0.000) difference also emerged across age groups relative to those reporting no symptoms, with most participants who were in the younger age groups (91%). More than half (41%) of those reporting no symptoms belonged to the 21-35 age group. The rest belonged to the ≤20 age group (20%), 36-50 age group (20%), and 51-65 age group (17%). Although the difference is not statistically significant, hospitalization due to severe symptoms and severe symptoms without hospitalization are side effects most reported by those in the ≤20, 21-35, and 36-50 age groups. Generally, side effects affect all age groups. Participants across different age groups uniformly report common symptoms. Since more participants belong to the younger age groups, most of those who reported no symptoms are younger participants, and more participants from the younger age groups also reported experiencing the most common symptoms as well as severe symptoms with or without hospitalization.

Table 4 compares the side effects reported by participants who got infected and who did not get infected with COVID-19 before vaccination. A highly statistical significance (*p*=0.002) emerged over the reporting of no symptoms from those who got infected and those who did not get infected with COVID-19 before vaccination. Most of those who reported no symptoms were not infected before vaccination (82%). Of those who reported side effects, more were also not previously infected before vaccination. Relative to the specific symptoms, a statistically significant difference emerged in only 2 side effects. Significantly more participants who got infected with COVID-19 before vaccination reported anosmia (68%, *p*=0.007) and ageusia (67%, *p*=0.011) as post-vaccination symptoms. Since more respondents were not infected before vaccination, the participants from this group reported no symptoms or experienced mild, moderate, and severe side effects. A statistically significant difference emerged in the 2 side effects reported more by those who got infected before vaccination.

**Table 4:** - Comparative vaccine side effects related to participants’ infection status before vaccination.

Infected before vaccinated	Yes	No	*P*-value
Hospitalized due to severe symptoms	10 (43)	13 (57)	0.655
Severe symptoms but not hospitalized	79 (33)	160 (67)	0.853
Long-lasting post-injection inflammation	50 (39)	79 (61)	0.410
Temporary post-injection inflammation	126 (26)	368 (74)	0.095
Fever	133 (33)	275 (67)	0.961
Musculoskeletal pain	148 (31)	335 (69)	1.000
Sore throat	25 (43)	33 (57)	0.342
Congestion	13 (33)	27 (68)	0.996
Diarrhea	11 (42)	15 (58)	0.814
Conjunctivitis	6 (43)	8 (57)	0.576
Headache	93 (31)	207 (69)	0.517
Anosmia	15 (68)	7 (32)	0.007
Ageusia	14 (67)	7 (33)	0.011
Difficulty in communicating	1 (10)	9 (90)	0.711
Difficulty in movement	35 (33)	72 (67)	0.993
Allergy and rash	6 (43)	8 (57)	0.199
Dyspnea	10 (28)	26 (72)	0.613
Chest pain	18 (42)	25 (58)	0.472
No symptoms	41 (18)	184 (82)	0.002
Total	834 (31)	1858 (69)	0.655

Values are presented as number and percentages (%). *P*-value of 0.05 was considered significant.

Table 5 shows the side effects relative to COVID-19 infection of the participants after vaccination. There is no statistically significant difference in the side effects reported by those who reported no COVID-19 infection after vaccination, COVID-19 infection after the first shot, and COVID-19 infection after the second shot. However, comparison across groups shows that even if most participants reported not having been infected with COVID-19 after vaccination with the first or second shot, they experienced vaccine side effects.

**Table 5:** - Vaccine side effects related to participants’ COVID-19 infection after vaccination.

Have you been infected with COVID-19 after receiving the vaccine?	No	Yes after the first shot	Yes after the second shot	*P*-value
Hospitalized due to severe symptoms	19 (83)	3 (13)	1 (4)	0.716
Severe symptoms but not hospitalized	215 (90)	18 (8)	6 (3)	0.976
Long-lasting post-injection inflammation	120 (93)	5 (4)	4 (3)	0.689
Temporary post-injection inflammation	454 (92)	31 (6)	9 (2)	0.987
Fever	369 (90)	31 (8)	8 (2)	0.973
Musculoskeletal pain	437 (90)	37 (8)	9 (2)	0.945
Sore throat	52 (90)	4 (7)	2 (3)	0.970
Congestion	34 (85)	5 (13)	1 (3)	0.704
Diarrhea	23 (88)	1 (4)	2 (8)	0.371
Conjunctivitis	14 (100)	0 (0)	0 (0)	0.852
Headache	269 (90)	26 (9)	5 (2)	0.738
Anosmia	20 (91)	2 (9)	0 (0)	0.959
Ageusia	20 (95)	1 (5)	0 (0)	0.963
Difficulty in communicating	5 (100)	0 (0)	0 (0)	0.975
Difficulty in movement	100 (93)	4 (4)	3 (3)	0.781
Allergy and rash	13 (93)	0 (0)	1 (7)	0.615
Dyspnea	34 (94)	1 (3)	1 (3)	0.915
Chest pain	38 (88)	4 (9)	1 (2)	0.976
No symptoms	214 (95)	8 (4)	3 (1)	0.358
Total	2450 (91)	181 (7)	56 (2)	

Values are presented as number and percentages (%). *P*-value of 0.05 was considered significant.

Table 6 shows the duration of the vaccine side effects reported by the participants. Statistically significant variances in the duration of certain symptoms emerged. Most of the symptoms lasted for 1 to 3 days or up to a week. Hospitalization due to severe symptoms lasted for 1 to 3 days (48%) or up to 1 week (26%) for most participants. Long-lasting post-injection inflammation lasted from 1 to 3 days (65%) or up to a week (29%) for nearly all participants. Temporary post-injection inflammation (86%) and fever (84%) lasted for 1 to 3 days for most participants reporting this side effect. Almost all participants who experienced musculoskeletal pain stated that it lasted for 1 to 3 days (70%) or up to a week (24%). Most participants who reported experiencing sore throat (90%), congestion (88%), and diarrhea (87%) stated that this lasted for 1 day up to a week. Most participants (79%) who experienced headaches did so for 1 to 3 days. More participants who experienced anosmia stated that the side effect lasted for 3 days to 1 week (45%) than those who experienced it for 1 to 3 days (32%). An equal number of participants (43%) who experienced ageusia did so for 1 to 3 days and up to 1 week. Most participants who experienced allergy and rash (79%) and dyspnea (80%) did so for 1 to 3 days or up to 1 week. An equal number of participants (37%) who experienced chest pain stated that this side effect lasted for 1 to 3 days or up to 1 week. Almost all participants (95%) who reported no symptoms experienced no side effects for 1 to 3 days, and only a very small number reported no symptoms in between the 2 doses. Most side effects waned within 3 days to 1 week after the first shot and there were almost none following the second shot.

**Table 6:** - Vaccine side effect duration between the 2 doses.

Symptom duration	A day to 3 days	3 days to a week	Week to a month	>3 months	*P*-value
Hospitalized due to severe symptoms	11 (48)	6 (26)	4 (17)	2 (9)	0.001
Severe symptoms but not hospitalized	184 (77)	43 (18)	11 (5)	1 (0.4)	0.667
Long-lasting post-injection inflammation	84 (65)	38 (29)	6 (5)	1 (1)	0.029
Temporary post-injection inflammation	384 (86)	55 (12)	7 (2)	2 (0.4)	0.001
Fever	363 (84)	55 (13)	13 (3)	3 (1)	0.001
Musculoskeletal pain	215 (70)	74 (24)	15 (5)	5 (2)	0.001
Sore throat	32 (64)	13 (26)	5 (10)	0 (0)	0.042
Congestion	19 (54)	12 (34)	4 (11)	0 (0)	0.004
Diarrhea	11 (47)	9 (40)	2 (9)	1 (4)	0.004
Conjunctivitis	9 (64)	3 (21)	1 (7)	1 (7)	0.207
Headache	236 (79)	52 (17)	11 (4)	1 (0.3)	0.362
Anosmia	7 (32)	10 (45)	2 (9)	3 (14)	0.001
Ageusia	9 (43)	9 (43)	2 (10)	1 (5)	0.006
Difficulty in communicating	4 (80)	1 (20)	0 (0)	0 (0)	0.962
Difficulty in movement	79 (74)	21 (20)	6 (6)	1 (1)	0.913
Allergy and rash	6 (43)	5 (36)	2 (14)	1 (7)	0.011
Dyspnea	16 (44)	13 (36)	6 (17)	1 (3)	0.001
Chest pain	16 (37)	16 (37)	9 (21)	2 (5)	0.001
No symptoms	213 (95)	6 (3)	2 (1)	4 (2)	0.001
Total	1897 (77)	441 (18)	108 (4)	30 (1)	

Values are presented as number and percentages (%). *P*-value of 0.05 was considered significant.

Overall, the participants regardless of age, gender, and infection status before vaccination reported the same common side effects. The most common symptoms for all vaccinated participants were temporary post-injection inflammation, musculoskeletal pain, fever, and headache, while the less common symptoms were conjunctivitis, allergy and rash, and talking difficulty. In this study more females than males experienced side effects. In addition, all age groups report common symptoms but the participants who reported no symptoms and who reported mild, moderate, and severe symptoms were mostly young, because more participants belong to the younger age groups. Since more participants were not infected before vaccination, most of those who reported no symptoms and experienced symptoms were not infected before vaccination. A statistical difference emerged in only 2 symptoms between those infected and not infected before vaccination. Also, there is no statistically significant difference in the side effects reported by those who reported no infection after vaccination, infection after the first shot, and infection after the second shot. Most side effects, mild, moderate, and severe, waned within 3 days to 1 week after the first shot and there were almost no side effects following the second shot.

## Discussion

COVID-19 vaccine caused side effects for most people. More than 90% of the respondents reported side effects (Table 1), which is much higher than the previously reported 25% rate of post-vaccination symptoms.^12^ Side effects are legitimate concerns that affect vaccine acceptability.^13^ Infection before vaccination does not influence the occurrence of post-vaccination side effects. Most of those who got infected or did not get infected before vaccination reported post-vaccination symptoms and common side effects. The most common symptoms for all vaccinated participants were temporary post-injection inflammation, musculoskeletal pain, fever, and headache while the less common symptoms were conjunctivitis, allergy and rash, and talking difficulty (Table 1). The commonly reported symptoms coincide with previous reports.^8,14-23^ However, the results of the study indicated that pre-vaccination infection may influence the onset of certain side effects, such as anosmia and ageusia reported more by those who got infected before vaccination and temporary post-injection inflammation and allergy and rash reported more by those who did not get infected before vaccination. Apart from informing vaccine recipients on the common side effects, it is also important to notify those who did not get infected and those who got infected before vaccination on specific side effects that they might experience. Gender influences post-vaccination side effects. Most male and female respondents reported experiencing vaccination-associated side effects. Male and female respondents also reported the same common side effects. However, gender affects the likelihood of experiencing post-vaccination symptoms. Males have a lower probability of experiencing any side effects, while females face a higher likelihood of experiencing side effects (Table 2). Gender may also determine the onset of certain side effects. More females than males experienced side effects, and more females experienced severe symptoms that require or do not require hospitalization (Table 2). The results align with previous studies linking gender to a greater probability of experiencing post-vaccination side effects and more severe symptoms but differed from prior research indicating that gender has no impact on the side effects.^18,20-23^ Although there is yet no consensus on the influence of gender on the onset and specific symptoms experienced after COVID-19 vaccination, the results of this study support the need to explain to the female population on the higher possibility of experiencing symptoms that may require or not require hospitalization. All age groups report common symptoms, but the participants who reported no symptoms and who reported mild, moderate, and severe symptoms were comparatively younger (Table 3). The results were partly similar to a previous study indicating that young people have a higher proportion of side effects and partly aligned with studies showing that age has no statistically significant impact on vaccine symptoms.^18,20-23^ More participants belonged to the younger age groups so more of those who reported no side effects were younger people and more of those who reported side effects of various severity were also younger people. Age does not seem to be a conclusive determinant of the onset of side effects or the occurrence of specific side effects.

Most of those who reported no symptoms and experienced symptoms were not infected before vaccination (Table 4). One previous study showed that a history of infection has no significant association with cutaneous side effects.^23^ The results of this study partly aligned with the previous study. Experiencing symptoms is more likely for those who were not infected before vaccination, as can be assumed as more people did not get infected before vaccination. However, a statistical difference emerged in the 2 side effects, with more of those who got infected before vaccination reporting anosmia and ageusia as symptoms (Table 4). There is no statistically significant difference in the side effects reported on Table 5. This study found no statistical association between the onset of side effects and infection before or after vaccination. There is no statistical difference in the symptoms reported by those who got infected before vaccination or after the first and second dose. It is important to clarify to vaccine recipients that a prior infection does not prevent the occurrence of vaccine symptoms.

Most side effects were mild or moderate, severe side effects waned within 3 days to 1 week after the first shot, and there were almost no side effects following the second shot (Table 6). This aligned with a previous study showing that COVID-19 vaccination has mostly minor side effects.^17^ The results of this study did not confirm a prior study indicating that side effects were more common after the second dose.^21^ Since concerns about side effects significantly affect vaccine acceptance, it is vital to let people know that they are likely to experience side effects but these side effects tend to be mild or moderate and only last up to a week.^13^

### Study limitations

The period between the vaccine doses were not collected, the residency of the participants and the number of people living in the same place, and how the contracting of the infection has occurred after the vaccination (if known to the participants).

In conclusion, concerns over side effects influence the decisions of people to get vaccinated. To ease these concerns, the public should be made aware that they are likely to experience at least one side effect, with temporary post-injection inflammation, musculoskeletal pain, fever, and headache as the most commonly reported side effects across the board. However, the common symptoms are mild to moderate and the side effects last for a short period for most people. People will experience side effects whether they were infected or were not infected before vaccination, but there are certain symptoms that more of those who were infected and not infected before vaccination will experience. Side effects disproportionately affect women. People experience side effects regardless of age. Prior infection does not prevent post-vaccination symptoms. Side effects wane after the second dose.
